# PROBLEMS AND ISSUES FOR HOSTS OF SOCIAL PARTICIPATION ACTIVITIES: A CASE STUDY FROM JAPAN

**DOI:** 10.1093/geroni/igae098.2957

**Published:** 2024-12-31

**Authors:** Mai Takase, Jun Goto

**Affiliations:** University of Tokyo, Tokyo, Japan; Tokai University, Hiratsuka, Kanagawa, Japan

## Abstract

Social participation is important for maintaining the health of older adults. In Japan, many recreational activities and hobbies are available for community-dwelling older adults for social participation. Meanwhile, little is known about the problems and issues that hosts face when conducting these activities. In this study, we aimed to clarify the problems related to hosting activities and possible measures that could be taken to alleviate these problems. Activity hosts providing activities for older adults in a community space in Chiba Prefecture (Japan) were approached. The activity content included musical events, movie, crafts, exercise, and cafés. This community space is operated by city’s Social Welfare Council and all of the activities are hosted voluntarily by citizens. Semi-structured interviews were conducted with ten hosts. All hosts were ≥ 65 years old and the average years of operation was 9 ± 2.7 (mean ± SD) years. As a result, nine out of ten hosts reported that the decline of members who supported the activity owing to aging and death was the major problem. Directly asking friends for help in the operation, receiving help from attendees, and decreasing the frequency of activities were measures that was taken to continue with the activities. The hosts also found it difficult to recruit younger generations who could become the next leaders of the activity group because of their lack of personal connections with the younger generation. Social Welfare Council mediating the connection between activity hosts and the younger generation who are interested in voluntary activities were anticipated.

